# Biopsy-Proven Fulminant Myocarditis Requiring Mechanical Circulatory Support Following COVID-19 mRNA Vaccination

**DOI:** 10.1016/j.cjco.2022.02.004

**Published:** 2022-02-13

**Authors:** Shingo Kazama, Takahiro Okumura, Yuki Kimura, Ryota Ito, Takashi Araki, Takashi Mizutani, Hideo Oishi, Tasuku Kuwayama, Hiroaki Hiraiwa, Toru Kondo, Ryota Morimoto, Tomoaki Saeki, Toyoaki Murohara

**Affiliations:** a Department of Cardiology, Nagoya University Graduate School of Medicine, Nagoya, Japan; b Division of Cardiology, Nagoya City East Medical Centre, Nagoya, Japan

## Abstract

A 48-year-old woman suffered from cardiogenic shock with fulminant myocarditis following the second dose of COVID-19 vaccine (mRNA-1273). Venoarterial extracorporeal membrane oxygenation and Impella support were essential in achieving hemodynamic stability. Endomyocardial biopsy revealed lymphocytic infiltration with predominant immunostaining for CD8- and CD68-positive cells. The left ventricular ejection fraction improved significantly after treatment with mechanical circulatory support. Myocarditis following COVID-19 mRNA vaccination may also occur in middle-aged women; it may be fulminant and require mechanical circulatory support. Although our results suggest the involvement of cytotoxic T lymphocytes and macrophages, further investigation is needed before these can be established as pathogenetic mechanisms.

## Résumé

Une femme de 48 ans a souffert d’un choc cardiogène accompagné d’une myocardite fulminante après avoir reçu la deuxième dose du vaccin contre la COVID-19 (ARNm-1273). L’oxygénation par membrane extracorporelle veino-artérielle et l’assistance par Impella ont joué un rôle essentiel pour atteindre la stabilité hémodynamique. Une biopsie endomyocardique a révélé la présence d’infiltrats lymphocytaires avec une immunocoloration prédominante pour les cellules exprimant CD8 et CD68. La fraction d’éjection ventriculaire gauche s’est améliorée considérablement après un traitement par assistance circulatoire mécanique. Des cas de myocardite peuvent également survenir chez des femmes d’âge moyen après l’administration d’un vaccin à ARNm contre la COVID-19; ils peuvent être fulminants et nécessiter une assistance circulatoire mécanique. Bien que nos résultats laissent croire à une participation des lymphocytes T cytotoxiques et des macrophages, une étude approfondie s’impose avant de pouvoir cerner les mécanismes pathogènes.

Vaccination is being widely implemented due to the COVID-19 pandemic. Some cases of myocarditis following COVID-19 mRNA vaccination have been reported^1^; however, the histopathologic and immunologic mechanisms by which this occurs remain unclear. We present a rare case of fulminant myocarditis in a middle-aged woman who received the second dose of a COVID-19 mRNA vaccine, and we consider the histopathologic findings of this case.

## Case

**Figure 1:**
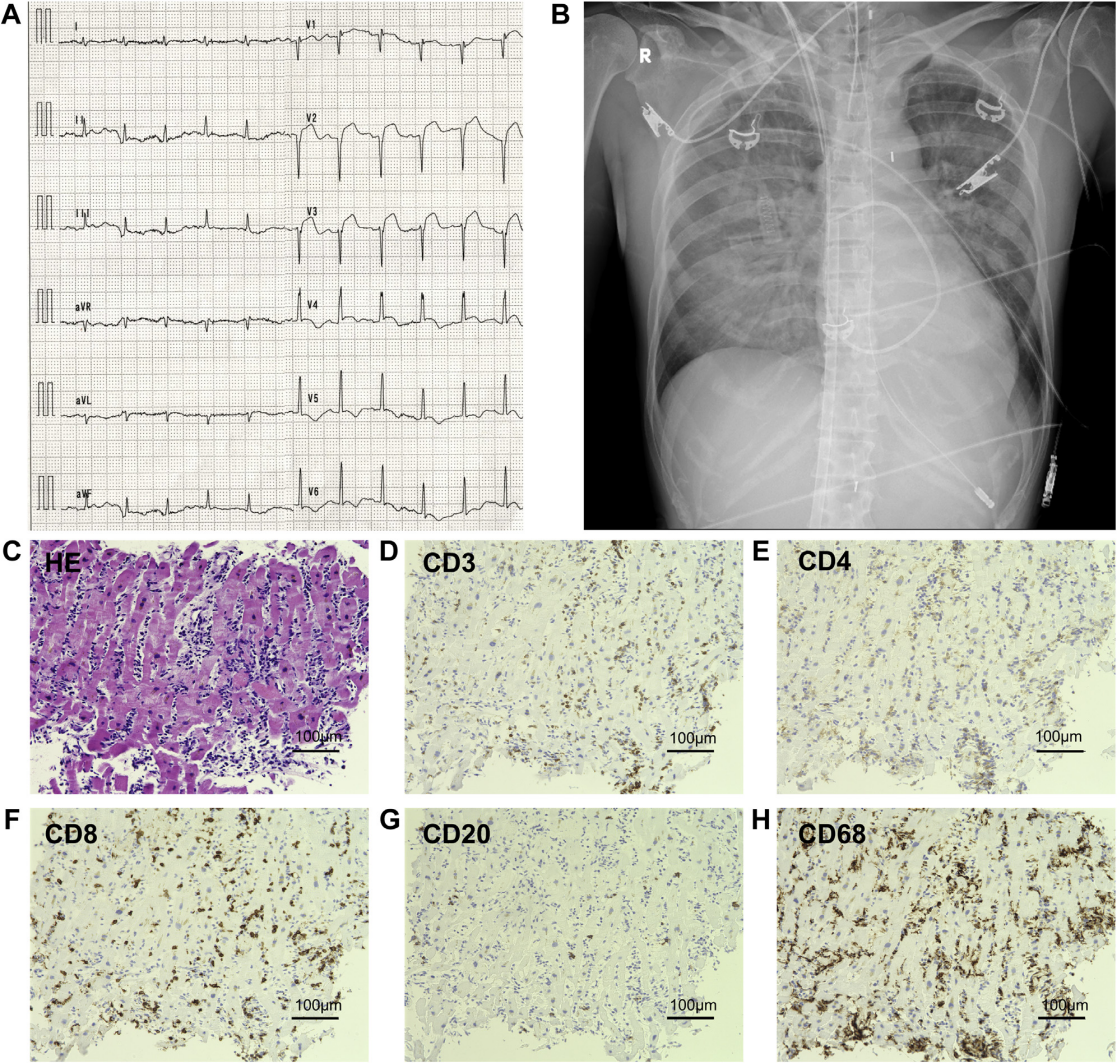
(**A**) Electrocardiography at the time of initial examination showing ST-segment elevation in the V1–V4 inductions. (**B**) Chest radiography at the time of transfer showing marked pulmonary congestion. (**C**) Hematoxylin and eosin (HE) staining of myocardial tissue showing lymphocyte infiltration. (**D**) Cluster of differentiation (CD)3 staining on immunostaining, suggesting infiltration by T lymphocytes. (**E, F**) CD8 staining (indicating cytotoxic T lymphocytes) is positive predominantly over CD4 staining (indicating helper T lymphocytes). (**G**) CD20 staining, indicating B lymphocytes, is negative. (**H**) CD68 staining suggesting marked macrophage infiltration.

A 48-year-old woman experienced persistent malaise for 1 week after receiving the second dose of the Moderna COVID-19 (mRNA-1273) vaccine; dyspnea appeared on the 7th day following vaccination. Her symptoms did not improve, and she was taken to the emergency department of her local hospital on the 9th day after vaccination. The second dose was administered 28 days after the first, and she had also experienced malaise after the first dose, which had resolved spontaneously. She had no significant past medical history and was postmenopausal. She had never experienced any previous side effects to the vaccine, and there was no history of autoimmune disorders in the patient or her family. She had been taking acetaminophen since vaccination, but she had not used any other drug. She presented with the following: body temperature of 36.1°C; blood pressure of 83/60 mm Hg; pulse rate of 113 beats/min; respiratory rate of 24 breaths/min; and saturation of 88% on 6L of oxygen. She was pale, with cold, clammy extremities. Laboratory tests showed multiple-organ damage and the following; lactate at 10.8 mmol/L (normal: 0.4-0.8 mmol/L); aspartate aminotransferase at 5358 U/L (normal: 13-30 U/L); alanine aminotransferase at 3079 U/L (normal: 7-23 U/L); lactate dehydrogenase at 4453 U/L (normal: 124-222 U/L); creatine kinase (CK) at 15,962 U/L (normal: 41-153 U/L); CK-myocardial band of 349 ng/mL (normal: < 5 ng/mL); and creatinine at 1.64 mg/dL (normal: 0.46-0.79 mg/dL). Troponin I and brain natriuretic peptide levels increased to 25.2 ng/mL (normal: < 0.026 ng/mL) and 1160 pg/mL (normal: < 18.4 pg/mL), respectively. Electrocardiogram showed ST-segment elevation in the V_1_–V_4_ inductions (Fig. 1A). Echocardiography showed a diffusely decreased left ventricular ejection fraction (LVEF) of 11%, and right ventricular contraction was also markedly decreased. Venoarterial extracorporeal membrane oxygenation (VA-ECMO) and an intra-aortic balloon pump (IABP) were immediately introduced with ventilatory support, owing to cardiogenic shock. The VA-ECMO cannula was inserted through the right femoral artery and vein, and the IABP was inserted through the left femoral artery. After establishing mechanical circulatory support (MCS), coronary angiography was performed, which demonstrated no significant stenosis. An endomyocardial biopsy (EMB) of the right ventricular septum was performed with the right jugular vein approach. A polymerase chain reaction test result for severe acute respiratory syndrome coronavirus-2 (SARS-CoV-2) was negative. The patient was transferred to our hospital for intensive care.

**Figure 2:**
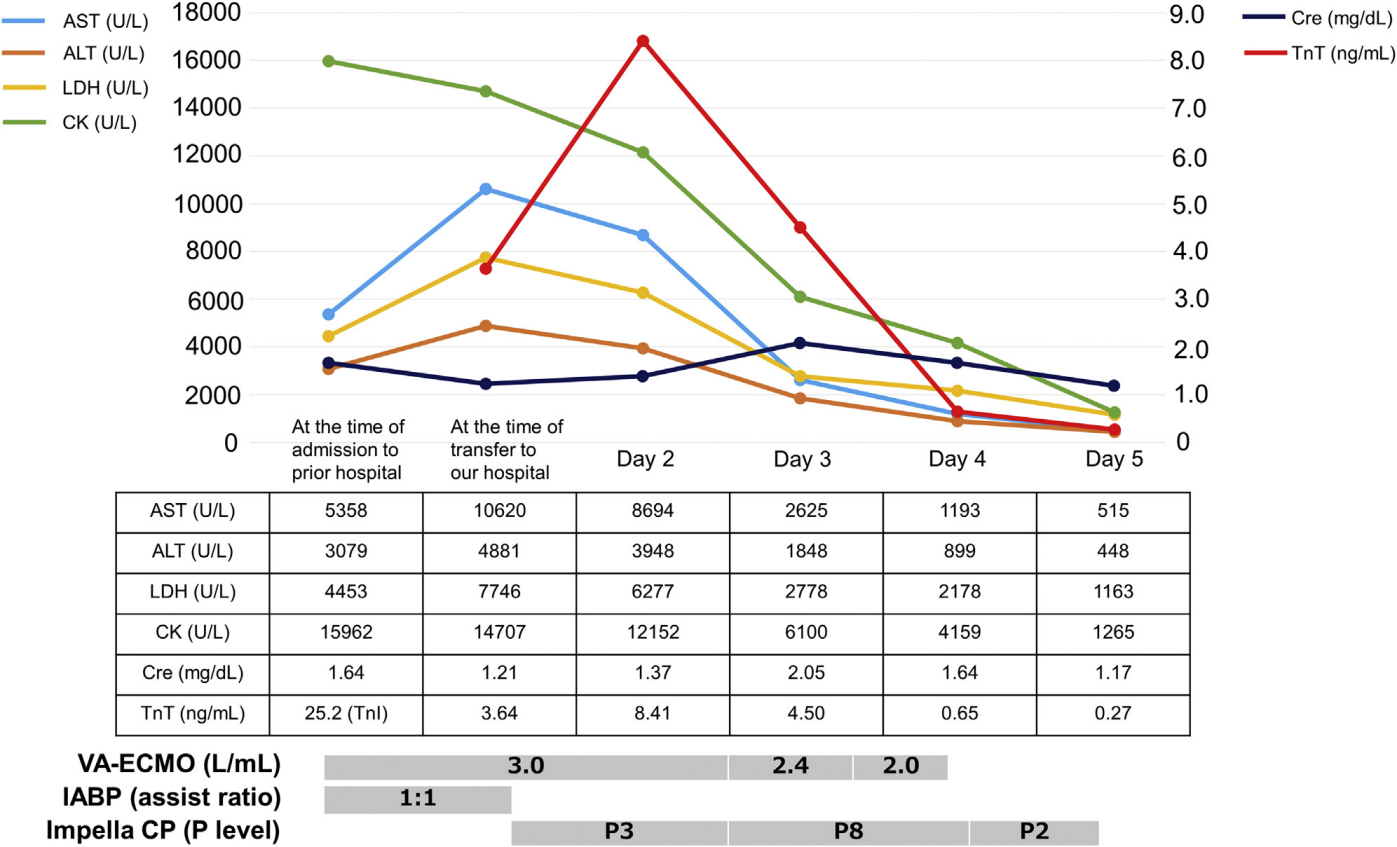
The graph shows the changes in laboratory data from admission at the prior hospital to the point of removal of mechanical circulatory support. (Troponin was measured as troponin I at the prior hospital.), ALT, alanine aminotransferase; AST, aspartate aminotransferase; CK, creatine kinase; Cre, creatinine; IABP, intra-aortic balloon pump; LDH, lactate dehydrogenase; Tnl, cardiac troponin I; TnT, cardiac troponin T; VA-ECMO, venoarterial extracorporeal membrane oxygenation.

On arrival, echocardiography showed severe left ventricular systolic dysfunction, with an LVEF of < 5.0% and persistent aortic valve closure (Video 1 , view video online), and chest radiography showed enhanced pulmonary congestion (Fig. 1B,), despite the use of dobutamine (5 μg/kg/min). Therefore, to unload the left ventricle and relieve pulmonary congestion, the IABP was changed to an Impella CP (Abiomed, Danvers, MA). After the introduction of the Impella CP, the dose of dobutamine was reduced to 2 μg/kg/min. On day 2 of hospitalization, although cardiac contractions had not fully improved, pulmonary congestion significantly improved, and laboratory test results showed signs of resolution of circulatory failure, with a lactate level of 1.2 mmol/L (Fig. 2). The histopathologic results of EMB performed at the previous hospital were confirmed; hematoxylin and eosin staining showed marked infiltration by inflammatory cells, mainly lymphocytes, and immunostaining showed significant cluster of differentiation (CD) staining (ie, CD3, CD 4, CD 8 [CD4 < CD8], and CD 68) (Fig. 1, C-H). Histopathologic findings showed no giant cells or frequent eosinophils; therefore, immunotherapy was not administered. On day 4, because LVEF improved to 32.6%, and the levels of CK, aspartate aminotransferase, alanine aminotransferase, and lactate dehydrogenase were also improving, VA-ECMO was discontinued (Fig. 2). On day 5, the Impella CP was removed, and the patient was extubated. On day 7, the patient was transferred from the intensive care unit to the general ward. Dobutamine was tapered off and discontinued on day 8. Echocardiography on day 17 showed that LVEF had improved to 59.8%, with no significant valvular disease (Video 2 , view video online). Additionally, cardiac magnetic resonance imaging (MRI) performed on day 21 showed no abnormalities, so we decided to monitor the patient carefully, without introducing cardioprotective drugs such as an angiotensin-converting enzyme inhibitor or a β-blocker. A polymerase chain reaction test of EMB specimens did not detect any viral genomes, such as adenoviruses, enteroviruses (including coxsackievirus and parvovirus), or human herpes virus. Paired serology also showed no significant increase in the levels of the antibodies of the abovementioned viruses. The patient underwent rehabilitation and was discharged on day 23 with no residual symptoms.

## Discussion

With the widespread use of COVID-19 mRNA vaccines, postvaccination myocarditis is attracting attention. This condition is conventionally known to be more common in young males under 30 years of age, and it is more common after the second dose than after the first; in many cases, these complications occur within a week of vaccination.^1^ Moreover, disease severity has been reported to be mild in most cases, and cases requiring MCS are very rare. Although the Lake Louise Criteria of cardiac MRI are often used as a noninvasive method of diagnosing myocarditis, the 2020 American Heart Association guidelines recommend EMB for diagnosing suspected cases of fulminant myocarditis requiring MCS, such as ours. Most of the reported cases of myocarditis following COVID-19 mRNA vaccination have been mild, and diagnosis is confirmed by cardiac MRI accordingly, but there are several histopathologic studies on myocarditis following COVID-19 mRNA vaccination.

In a large Israeli cohort of approximately 5.1 million people given the COVID-19 vaccine, 142 people were diagnosed with myocarditis after receipt of the BNT162b2-mRNA vaccine.^2^ EMB samples obtained from only 2 people showed foci of endo-interstitial edema and neutrophils, along with macrophages and lymphocytes with no giant cells. In the histopathologic pictures of 2 recently reported cases of post–COVID-19 vaccination myocarditis, infiltration by T lymphocytes and macrophages was visible, and B lymphocytes and plasma cells were also seen.^3^ Lim et al. reported fulminant myocarditis requiring VA-ECMO following the first dose of the BNT162b2-mRNA vaccine, in a woman^4^; in this case, histopathologic examination of EMB samples revealed marked and diffuse lymphocytic infiltration of the myocardium. Additionally, in a case report of fulminant myocarditis with systemic hyperinflammation after COVID-19 vaccination, an EMB showed cardiomyocytes with minute foci of cytoplasmic vacuolization and rare interstitial lymphocytic infiltrates.^5^ Histopathology of the myocardium in our case showed inflammatory cell infiltration, mainly lymphocytes, with some cytotoxicity. Immunostaining showed that the staining for CD8 was more positive than that for CD4; moreover, the staining for CD68 was positive, suggesting that cytotoxic T lymphocytes and macrophages may be largely involved in myocardial inflammation. In a case report showing the histopathology of fulminant lymphocytic myocarditis (not following COVID-19 vaccination), the myocardium was infiltrated with CD8-dominant T lymphocytes and macrophages, similar to our case.^6^ CD3 and CD68 positive findings are one of the hallmarks of immunostaining for lymphocytic myocarditis, but they are not disease-specific. However, T lymphocytes and macrophages are presumed to be involved in the pathogenic mechanisms. To date, no histopathologic findings have been established to distinguish myocarditis associated with COVID-19 vaccine from myocarditis that is not associated with COVID-19 vaccine.

Many reports have been made on COVID-19 infection and myocardial injury. In previous reports, the proposed mechanisms of myocardial injury are direct damage to the cardiomyocytes, systemic inflammation, myocardial interstitial fibrosis, interferon-mediated immune response, coronary plaque destabilization, and hypoxia. Whether myocardial injury has a common mechanism via the mRNA vaccine vs via COVID-19 infection is currently unclear. A recent meta-analysis speculated that myocarditis may develop when the immune system detects genes in the vaccine as antigens, thereby activating proinflammatory cascades and immune pathways.^7^ Other possible mechanisms include induction of cytokine expression via anti-idiotypic cross-reactive antibodies in the myocardium and abnormal induction of apoptosis leading to inflammation of the myocardium and pericardium.^7^ In addition to age and gender, certain genetic predispositions have been speculated to be risk factors for the development of myocarditis, but the details are unknown. Furthermore, the clinical course of fulminant myocarditis following COVID-19 vaccination also has not been established. The patient in this case was able to be weaned off MCS in a relatively short period of time, even though the patient's condition was initially lethal. Previous reports have suggested that left ventricular unloading with an Impella suppresses inflammatory cell infiltration in fulminant myocarditis.^8^ We speculated that, also in this case, left ventricular unloading by early introduction of an Impella CP may have suppressed inflammatory cell infiltration, and improved left ventricular contraction in a relatively short period. Therefore, early introduction of MCS with left ventricular unloading is important in fulminant myocarditis. The exact mechanism is still unclear. and more cases need to be accumulated; however, this report helps confirm that classic lymphocytic fulminant myocarditis can be triggered by a COVID-19 mRNA vaccine.

## Conclusions

Fulminant myocarditis following COVID-19 mRNA vaccination may occur in middle-aged women, and early introduction of MCS is important in severe cases. Although cellular immune mechanisms may be involved in the pathogenesis, further investigations are needed before establishing the pathogenetic mechanisms of COVID-19 vaccine–related myocarditis.

**Novel Teaching Points**

• Fulminant myocarditis following COVID-19 mRNA vaccination may occur in middle-aged women.
• Early introduction of MCS is important in fulminant myocarditis.
• Cellular immunity may be involved in the pathogenesis of this disease.

## Supplementary Material

Video 1 Echocardiography at the time of transfer to our hospital.

Video 2 Echocardiography on day 17.
